# Methodological innovations in studying marginalized populations in research on neurodegeneration and ageing

**DOI:** 10.3389/fnagi.2026.1823638

**Published:** 2026-07-15

**Authors:** Anja K. Leist, Eva Teyssier, Jure Mur

**Affiliations:** 1 Institute of Gerontology, Heidelberg University, Heidelberg, Germany; 2 Institute for Research on Socio-Economic Inequality, University of Luxembourg, Esch-sur-Alzette, Luxembourg

**Keywords:** biological ageing, brain ageing, cognitive ageing, dementia, marginalized groups, methodological innovations, social determinants of health (SDOH)

## Abstract

Underrepresented populations, often characterized by minority ethnicity, sex/gender or low socioeconomic status, have a higher risk burden and more adverse lifetime experiences of conditions negatively impacting cognitive health than better represented populations. There are several methodological challenges of research in and with marginalized populations that hinder research on improvements to their cognitive health. This review aims at presenting challenges of recruitment, data collection, and statistical analysis, and points to innovative methods for alleviating these challenges. Careful recruitment and data collection can ensure that marginalized populations are sufficiently represented in research. Multiple factors, including the clinical presentation of symptoms, accelerated biological ageing, and early life adversity need to be incorporated into statistical analysis. Innovative methodologies in research on neurodegeneration and ageing can improve the accuracy of descriptive, predictive and causal investigations, and at least partially address data inequity. These methods can contribute to the aims of reducing inequities in the distribution of healthcare and social care resources.

## Introduction

Health is an unequally distributed good. People of lower socioeconomic status, those from minority ethnic backgrounds and with other characteristics associated with low social prestige and power are at increased risk of mortality and morbidity. There are ethnic disparities in incidence of dementia in the U.S. (Mayeda et al., 2016), and education, income, and other socioeconomic indicators influence incidence and prevalence of dementia (Walsh et al., 2025). Ethnic differences in diagnosis, treatment and survival in Parkinson’s Disease have been documented and seem to intersect with neighborhood deprivation (Xie et al., 2021). For the neurodegenerative disease amyotrophic lateral sclerosis, ethnic differences are documented for treatment and healthcare utilization (Kuo et al., 2025). However, some studies point to higher incidence of neurodegenerative diseases, such as Parkinson’s Disease, in higher socioeconomic strata (Li et al., 2009), possibly reflecting higher healthcare utilization or earlier diagnosis in those groups. Overall, inequities in neurodegenerative disease and ageing are not well understood, as methodological challenges hinder accurate estimations of health differentials in neurodegenerative disease.

Marginalized populations, for the purpose of this review, are social groups that are less often in positions of power and privilege, have fewer resources, and who may face deprivation, discrimination or oppression. They may also disproportionally experience trauma, violence, or armed conflict. These groups can be characterized by ethnic minority status, low socioeconomic standing, or a certain sex, gender identity, or sexual orientation (Krieger, 2001, 2024). Experiences of discrimination and marginalization are shared widely by women in gender-unequal settings (Ribeiro et al., 2023). Other characteristics include visible or invisible disabilities, homelessness, and psychiatric or substance use disorders.

This review first outlines select neuroscience- and marginalization-relevant phenomena, continues with an explainer of methodological challenges, before presenting methods to increase participation of marginalized groups in research and statistical approaches to alleviate or overcome central research challenges. We conclude with observations about how research ecosystems can accommodate equitable research on neurodegeneration and ageing.

## Phenomenological considerations

Investigating neurodegeneration and ageing in marginalized groups requires ensuring assumptions and metrics across all observed populations. The clinical presentation of neurodegenerative disease can vary with language, literacy and other factors influencing cognitive assessment. The sets of risk factors and associated burden may differ, leading to changes in disease onset and trajectories.

### Early life adversity: accumulation of disadvantage affects brain structure and function

Stress is a psychological concept that describes exposure to situations that are commonly perceived as adverse and that challenge psychological and often also physiological needs, e.g., secure attachment, perceived safety, or appropriate nutrition. Adopting epidemiological perspectives, research has investigated stress for its potential to explain later-life brain structure and functioning. Biological mechanisms of stress are not fully elucidated, and stress may dysregulate different hormonal, inflammatory, or even gut microbial pathways during different life stages. However, a large body of literature has confirmed associations between different measures of stress and older-age brain markers and cognitive functioning. Prenatal stress leads to reduced hippocampal volume and learning impairments in adulthood, stress during childhood and adolescence exhibits persistent associations with behavior, stress during adulthood is associated with depression – a risk factor for dementia – and stress during old age is associated with lower hippocampal volume and lower memory scores (Lupien et al., 2009). Similarly, childhood community disadvantage is associated with lower brain volume in older age (Peterson et al., 2024). In studies that focus on old age, there is evidence for associations between early-life adversity and cognitive impairment and dementia, with animal studies suggesting causal relationships (Hackman et al., 2010; Short and Baram, 2019).

The theory of accumulation of disadvantage (Dannefer, 2003) is another relevant conceptual framework that can explain differences in experienced adversity between marginalized and non-marginalized groups over the life course. Distinct socioeconomic conditions and life-course opportunities often create fundamental differences between marginalized and non-marginalized groups. This results in a lack of overlap in characteristics that explain differences in outcomes, (a.k.a. lack of common support), which precludes successful matching between exposure groups (Hill and Su, 2013). This challenge may be partially addressed by documenting group differences via descriptive analysis and implementing study designs that stratify analyses for each social group. Where explicit considerations of marginalization are represented in the data, statistical matching is advised to increase comparability between exposure groups. This approach is now well established in epidemiology (Stuart, 2010). However, variables that capture marginalization status are often unmeasured (see below) and no quantitative data may fully capture the lived experience. Statistical adjustments for marginalization status and trauma are thus necessarily imperfect.

### Marginalization affects biological ageing

Ageing processes may arise earlier in traumatized, vulnerable, or deprived groups. Understanding the ageing process thus requires a stronger focus on younger and middle-aged populations. Some evidence on accelerated ageing for socioeconomically disadvantaged groups exists, for instance in individuals with higher levels of neighborhood deprivation (Lei et al., 2019; Ribeiro et al., 2022a,b). Using a capacity-based approach to measure healthy ageing (Menassa et al., 2023), research in a Brazilian sample found that, among other sociodemographic determinants, individuals self-identifying as mixed race (compared to those self-identifying as White) and with no schooling or fewer than four years of education, were less likely to experience healthy ageing (Ribeiro et al., 2026). Reiterating the importance of early-life adversity mentioned above, healthy ageing was also less likely for those reporting poor health and experiencing hunger during childhood (Ribeiro et al., 2026).

Other research has shown that accelerated ageing, sometimes referred to as “weathering,” may be affecting groups impacted by social adversity, i.e., low socioeconomic status, more strongly as evidenced for instance in peripheral blood DNA methylation measures (Fiorito et al., 2017). Similar patterns have been found for socioeconomic and race/ethnicity gradients in immunosenescence (Noppert et al., 2023). Accelerated ageing may thus also affect minoritized groups more strongly on other health-related outcomes, and cause an earlier onset of menopause in Black women, albeit difficult to identify as selection bias at enrolment masks the disparity (Reeves et al., 2023). Similarly, ethnic differences in the onset of hypertension, insulin resistance, and diabetes – all modifiable risk factors for dementia – are even larger when taking study selection into account (Reeves et al., 2022).

Concerning socioeconomic differences, recent research has shown that phenotypic ageing is partly socially stratified (Alonso-Perez et al., 2026), and first evidence has been emerging that epigenetically measured ageing, particularly for those measures that are trained on physiological function, is socially stratified (Needham et al., 2025).

Accelerated ageing in marginalized populations likely translates into premature cognitive ageing. Individuals in lower-resource settings, often proxied with country levels of economic development, i.e., in lower- and middle-income countries, show a higher prevalence of mild cognitive impairment and dementia compared to higher-resource settings, and low education and rural residence are even more strongly associated with risk of cognitive impairment and dementia (Ribeiro et al., 2022a,b).

### Clinical ascertainment of neurodegenerative disease

Cognitive decline occurs across most of the adult life course, both in people that eventually develop dementia and in those that do not. Severe cognitive impairment can be thought of as falling below a threshold under which every-day functioning is too low for independent living. Factors associated with marginalization can affect both the baseline and the steepness of its slope. Thus, the above threshold will, on average, be reached sooner for people from marginalized groups than for the majority.

It is difficult to evaluate the clinical presentation of cognitive impairment without considering the levels of education, general literacy, health and digital literacy, and lifetime experiences modulating cognitive stimulation. Schooling quality is a relevant determinant to increase cognitive skills, and populations with lower schooling quality have fewer opportunities for cognitive development than populations with more schooling resources (Lövdén et al., 2020; Seblova et al., 2023).

Cognitive tests often “correct” for factors associated with cognitive function, e.g., age, education, and ethnicity to yield norms for the threshold of diagnosis for different subgroups. In practice, this means that individuals characterized by a certain risk factor are evaluated in comparison to people with that same risk factor as opposed to the general population. For example, education is known to affect cognitive development (Lövdén et al., 2020) and using education-adjusted norms to evaluate cognitive impairment could prevent false positive diagnoses that are in fact attributable to lower baseline cognitive status. Neuropsychological assessment is generally considered as context-dependent regarding populations and socioeconomic settings. In the absence of robust cross-setting diagnostic assessment, norms adapted for different racial/ethnic groups (Manly et al., 2022) aim at accounting for social structures and differential life-course opportunities related to the development of cognitive functioning of ethnic minority groups. However, this often results in poorer discriminative accuracy for a diagnosis of dementia, due to adjustments for common causes of cognitive scores and cognitive status (Piccininni et al., 2023). Marginalized groups already experience higher morbidity (Mayeda et al., 2016), lower utilization of and higher unmet needs for healthcare (Horner-Johnson and Dobbertin, 2014) and social care (Lin and Liu, 2024) than the majority population. Thus, norms in cognitive testing might increase the threshold at which cognitive impairment is diagnosed and further entrench existing inequalities. Conversely, misdiagnosis of dementia may entail negative consequences such as stigmatization, job loss etc., It is advised to run additional diagnostics for dementia ascertainment to increase accuracy; however, we currently lack adequate, multimodal diagnostics globally (Gauthier et al., 2021).

### Selective survival

Life expectancy of marginalized groups differs from that of the majority when stratified by socioeconomic status in Europe (Mackenbach et al., 2019), by county, race and ethnicity in the United States (Dwyer-Lindgren et al., 2022), and across Latin American cities (Bilal et al., 2019). Differences in life expectancy reach several years and are even more dramatic for other, extremely marginalizing conditions such as homelessness (Nusselder et al., 2013). Similarly, traumatizing experiences, notably armed conflict, war, and starvation, lead to substantial differences in health and mortality.

Aside from these phenomenological considerations which are often not fully measurable, additional methodological challenges pose barriers to research on marginalized populations in brain ageing. These are outlined in the following section.

## Methodological challenges related to data collection

Methodological challenges arising in research with underrepresented communities concern information about marginalization status, biases in enrolment and attrition (Rojas-Saunero et al., 2023a), and outcome ascertainment.

### Selection bias in enrolment

Early in the research pipeline, researchers and funders launch research programmes, formulate research questions, and decide on communication channels and means of sample recruitment (Chen et al., 2021). Geographical distance, limited availability of public transport, and long assessment times may conflict with work schedules or care duties of some participants, presenting participation barriers which are usually higher for marginalized groups. Selective survival may occur before enrolment (Mayeda et al., 2018). Neurodegeneration and ageing studies often require structural brain measures obtained through imaging, but the above barriers impede such research in marginalized groups. Ethnic minority groups may be more reluctant toward biomarker collection than non-minority groups, albeit socioeconomic indicators seem similarly relevant to understanding the willingness toward participation in research (Erickson et al., 2022; Gooding et al., 2025). Reliable brain-wide association studies that relate brain-imaging markers to cognitive phenotypes namely require 1000s of participants to ensure reproducibility of effect estimates (Marek et al., 2022). Increased selection bias in imaging sub-datasets (Bradley and Nichols, 2022) of already selective datasets such as UK Biobank poses an additional challenge for equitable brain ageing research (Marek et al., 2022).

### Incomplete information about possible marginalization status

How are social groups defined in the research question and how are relevant characteristics operationalized? Information on ethnicity is often lacking, e.g., in the electronic healthcare record (EHR) or in population-based surveys. Empirical differences in how the healthcare system is navigated and the extent to which respondents perceive benefits after healthcare contact can only be quantified with this information (Paccoud et al., 2022). Similarly, gender identity (as an additional category to sex assigned at birth) is often not assessed. However, gender identity significantly associates with health, with non-binary individuals reporting higher modifiable risk burden for neurodegenerative diseases, e.g., related to cardiovascular events and depression (Brady et al., 2024).

### Selective attrition

Attrition, i.e., loss to follow-up, can also amplify selection issues. Study attrition may be due to selective survival but also limited time and resources to participate in research, which across multiple cohort or panel studies is more prevalent among participants from lower socioeconomic strata. A related methodological problem is competing risk of death which limits our ability to detect the risk of e.g., dementia due to death occurring before onset of clinical symptoms of the disease and challenges the estimation of cognitive decline (Davis-Plourde et al., 2022). Competing risk of death can – depending on the question – bias causal inference investigations (Rojas-Saunero et al., 2023b) and reduce the predictive capacity in marginalized groups. It is important to systematically investigate possible causes of bias by documenting socioeconomic gradients in attrition and their potential implications for the generalizability of the findings.

### Incomplete outcome ascertainment

Marginalized and non-marginalized groups differ in healthcare and long-term care utilization. This holds independently of economic access and with some intersections with gender, e.g., Hispanic women are less likely than Hispanic men to use nursing home care (Dunlop et al., 2002). Health systems may also inadvertently draw barriers for underrepresented communities which reduces potential health benefits (Hui et al., 2020). Such reduced healthcare contact may preclude the diagnosis of existing neurodegenerative conditions. Increased healthcare utilization, for example, increases the probability of being diagnosed with dementia (Mur et al., 2024). Similarly, research on injurious falls shows that the risk of dementia is elevated in the year after a fall, particularly in the first 1 to 2 weeks (Ordoobadi et al., 2024). This suggests that increased healthcare utilization following a fall leads to the detection and diagnosis of dementia. Differential healthcare use by ethnic background indicates that in large cohorts, undetected neurodegenerative disorders may be more common in marginalized populations.

Some methodological challenges are addressable through improvements in data collection with marginalized groups, detailed in the next section.

## Improving participation of marginalized individuals in research through increased outreach efforts

To improve the representation of marginalized individuals in datasets and thus increase data equity, efforts are necessary to connect with the relevant communities and build trusting relationships. Ideally, this should be done before commencing recruitment to the study (Brewster et al., 2019). In the following we list different ways to increase enrolment and retention of underrepresented groups.

Linguistic, economic, social, or geographical barriers to participation need to be removed. The use of digital assessments may remove barriers of geographical distance and reluctance to e.g., visit assessment centers or provide sensitive information in face-to-face interviews. Conversely, digital tools may obstruct individuals with low digital literacy. Sex/gender differences in the readiness to use digital tools in older adults have been documented (Van Elburg et al., 2022), but such differences are rarely reported across other sociodemographic groups.

Strategic framing and destigmatizing language improve trust and participation, especially on sensitive topics. Neutral study titles reduce stigma and increase responsiveness. For instance, participants may avoid a “questionnaire on drugs” but accept a “health survey.” Similarly, investigations on homelessness rely on respectful terminology (Castañeda and Smith, 2023). In research on neurodegeneration, framing a study around “healthy ageing” or “memory” rather than “dementia” can reduce anxiety and promote recruitment. Aside from careful wording, increased efforts such as repeated engagements with potential participants and using multiple communication channels are necessary to reach, for instance, lower-educated individuals with migration background (Teixeira-Santos et al., 2025). Trust in research by potential participants develops through researchers and research institutions, consistently and over a longer time frame, engaging with minority communities, while considering deeper issues of systemic justice and equity (Gilmore-Bykovskyi et al., 2022).

Specific studies aimed at recruiting marginalized groups in observational studies, such as the Minority Aging Research study (Barnes et al., 2012) and the SABRE study (Tillin et al., 2013) can improve our knowledge of brain health in underrepresented groups. Other large cohort studies put efforts in increasing enrolment of participants from ethnic minorities and systematically compare racial/ethnic and socioeconomic differences of their cohort to the general population, such as the Wisconsin Registry for Alzheimer’s Prevention (Ma et al., 2025). This applies equally to women, given the historical neglect in research. Studies like SWAN address previously neglected phases of life, including the menopausal transition (El Khoudary et al., 2019). Low-educated individuals are less likely to be recruited and participate in clinical trials (Lazaar et al., 2025). To preferentially enrol low-socioeconomic status and minority-ethnicity individuals, some large trials have implemented screening algorithms (Baker et al., 2024; Van Der Endt et al., 2025). Other trials have increased recruitment efforts for minority-ethnicity participants (Johnson et al., 2020; Yaffe et al., 2024). However, these efforts need to be expanded to arrive at more data on marginalized populations. Where governmental funding has been cut, philanthropists and foundations are called upon to continue this important research agenda. Funders and institutions, at the beginning of the ethical pipeline, shape the extent to which research into health equity can be realized.

In the past few years, some new methodological developments have improved the possibilities for research with marginalized populations despite incomplete data. These will be outlined in the following section.

## Methodological innovations to address challenges in research with marginalized populations

We present the methodological innovations according to the three main purposes of research in the social and health sciences: description, prediction, and causal inference (Hernán et al., 2019). Mapping these specific goals to methodologies can alleviate data quantity and selection issues (Leist et al., 2022).

Descriptive goals like the estimation of area-level disease burden are increasingly attainable using predictive machine learning (ML) algorithms. These algorithms, when trained on other external datasets enable the estimation of the quantity of interest in the target population even when data on the outcome of interest are missing (Luo et al., 2015). Provided that some associations between basic sociodemographic characteristics and health outcomes are available in a training dataset, this method requires available sociodemographic data to estimate unavailable disease burden in another population. This could be applied to support descriptive epidemiology in data-poor settings. However, more detailed sociodemographic information will provide more fine-grained estimations.

**FIGURE 1:**
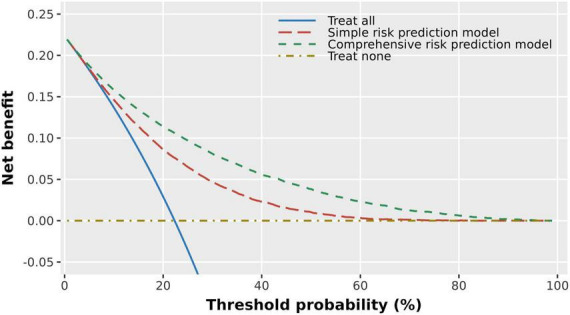
Example of a decision curve for a hypothetical medical intervention. Traditional performance metrics, such as the area under the receiver operating curve, sensitivity, specificity, etc., provide an assessment of discriminative accuracy of a prediction model (ability to differentiate between cases and non-cases) but insufficient insight into its clinical utility. An intuitive but underutilized approach to assess clinical utility is decision curve analysis (DCA) (Vickers and Elkin, 2006). DCA uses the concept of threshold probability *p*_*t*_, to designate the probability at which the expected benefit of a clinical intervention is equal to the expected benefit of avoiding the intervention. For example, let’s imagine that a clinician was assessing the appropriateness of prescribing a medical intervention to prevent or treat a disease. If she judged that the harms due to a missed opportunity to prevent the disease were nine times greater than a potentially unnecessary medical intervention, the *p*_*t*_ would equal 10%. This implies that for a patient whose risk of disease is 10%, the expected benefit of treating equals that of not treating. The exact value of *p*_*t*_ varies from case to case and depends on the clinical picture, and the subjective values of both clinician and patient. The second component of DCA is the notion of net benefit: a metric that places the benefits and harms of a decision on the same scale. DCA can be used to plot the net benefit of a prediction model across a range of likely values for *p*_*t*_. For example, if barely any clinician prescribed the intervention if the risk of disease was ≤ 5%, but almost all clinicians prescribed the intervention if the risk was ≥ 30%, net benefit could be plotted for values of *p*_*t*_ between 5% and 30%. The net benefit of using a clinical prediction model would then be compared with two default strategies: intervene on everybody and intervene on nobody. Given her threshold probability for the intervention, the clinician could use DCA to assess whether the use of the prediction model was useful when compared to the default strategies. The figure shows an example decision curve for a hypothetical medical intervention. The net benefit of intervening on everybody and of intervening on nobody are compared with the net benefit of either using a simple prediction model or a more comprehensive risk prediction model to guide the decision of whether to perform the intervention. In this example, the use of the risk prediction model is superior to the three alternative strategies between the threshold probabilities of 5% and 90%. Note that the entire x-axis is shown here; normally, the x-axis would be restricted to the range of relevant threshold probabilities.

For prediction – the diagnosis and prognosis of health events - multiple risk prediction algorithms for dementia exist, but many lack validation in clinical practice (Brain et al., 2024). Further, the algorithms’ ability to increase the net utility of concrete clinical decisions (Figure 1) is rarely analysed. Here, methodological innovations could improve estimation accuracy in small samples and account for lower data quantity.

Transfer learning offers a robust solution to address data scarcity and improve accuracy for less represented groups if datasets can be combined (Gao and Cui, 2020). This method combines statistical insights from high-accuracy but low-quantity data and low-accuracy high-quantity data. By “learning” refined coefficients from the smaller sample, algorithms can be tailored to improve prediction accuracy for small sub-samples (Kim et al., 2025). Collecting new data with this method in mind may be particularly suitable in resource-poorer environments where a small sample with clinical ascertainment may be combined with a larger sample with more cost-efficient data collection, e.g., through an online questionnaire.

BOX 1 Addressing selection and attrition bias through inverse-probability weighting. Inverse probability weighting (IPW) is a statistical method used to improve the representativeness of a database in case of imbalanced sampling between different sub-populations (Robins et al., 2000). Selection bias is a pervasive problem in volunteer datasets and weights to alleviate this bias can be constructed or are ready-for-use for some cohorts, e.g., the UK Biobank (Van Alten et al., 2024). A non-representative sample can occur at several stages of the data collection pipeline. In longitudinal studies for instance, both selective enrolment and selective attrition and mortality can introduce bias (Brayne and Moffitt, 2022). A participant needs to be made aware of the existence of a study, agree to participate, and remain in the sample until data collection is complete. Participants with different characteristics may be more or less likely to be recruited into and stay in the study, thus creating an imbalance between groups of participants. IPW can alleviate this imbalance by creating a “pseudo-population” representing the original target demographic. When different reasons for exclusion exist, it is possible to combine different weights. Let two sub-populations, A and B, be equally sized in the real world. However: Group A is highly compliant; most agree to join and stay in the study. Group B is harder to reach; many refuse participation or drop out. In the final dataset, group A will be overrepresented and group B scarce. To correct this, we assign a higher “weight” to members of group B, allowing them to “fill-in” for their missing peers. The weight is calculated as the inverse of the probability of being included or remaining in the sample based on participant characteristics (e.g., ethnicity, health status, wealth). When different participation and attrition rates exist, weight can be multiplied to create a final combined weight. While powerful, IPW requires careful application. First, broad categories can be dangerous in heterogeneous sub-populations. Inflating the weight of a specific demographic (e.g., highly educated migrants) to represent a diverse minority group may increase bias (Brayne and Moffitt, 2022). Thus, a nuanced understanding of the sample composition is vital. Moreover, extremely low probabilities yield massive weights, potentially destabilizing the analysis. Methods to “stabilize” weights, such as using marginal probabilities, can mitigate this (Robins et al., 2000). Finally, IPW assumes missing data is explainable; it cannot correct for unobserved factors or pure randomness.

Prediction models often show differential performance, meaning that some group may benefit less or even be harmed by their use. Since healthcare algorithms are frequently trained on biased data, they can reflect and perpetuate systemic inequalities (Vyas et al., 2020). Furthermore, the lower explainability of ML-derived algorithmic decision making compared to traditional scoring can magnify these existing biases (Chen et al., 2021). Mitigating these issues requires employing richer, multi-modal datasets (Wang et al., 2023) and explicitly incorporating the differential needs of social groups into algorithmic design (Coots et al., 2025). Bias mitigation strategies have been reviewed previously (Cary et al., 2023).

Ultimately, translating the research on brain ageing into equitable clinical practice is challenging. Even when an intervention’s effects are known for the general population, treatment benefits and foregone-treatment losses may be substantially different in marginalized groups. For instance, standard risk factors (like genetic) are seemingly less predictive of dementia in African Americans (Rajan et al., 2019). Here, net utility analysis (Figure 1) needs to further incorporate heterogeneous subgroup effects. To improve decision making for underrepresented groups, we need sufficient data on treatment preferences and effectiveness.

Causal inference presents unique challenges in the context of brain ageing research. Randomized Controlled Trials (RCT) are often infeasible due to the high costs of long follow-ups. Moreover, manipulating putative causes (exposures) is unethical because they negatively affect other domains of health (Hernán and Robins, 2016; Hilton Boon et al., 2021; Imai et al., 2023; Kim et al., 2023). Causal inference methods for observational data, such as target trial emulation (Hernán and Robins, 2016), regression discontinuity designs (Hilton Boon et al., 2021), and difference-in-difference methods (Imai et al., 2023; Kim et al., 2023) can be used when RCTs are not possible.

However, successful emulation requires high-quality data with complete ascertainment from both marginalized and non-marginalized groups (Mur et al., 2024). A case-control design can represent an alternative approach for clinical interventions with small samples. Here researchers recruit a small sample from the population of interest and compare cases with matched controls drawn from large datasets, such as electronic health records.

Regardless of aim, a sample that contains data from underrepresented groups likely has a high proportion of missing data in domains crucial to the marginalization status, such as early-life adversity, socioeconomic status and other social determinants of health. Because this data might not be missing completely at random, complete-case analyses can lead to bias. In such cases, multiple imputation can reduce bias and increase estimation efficiency (Hughes et al., 2019; White and Carlin, 2010). The use of m-DAGs – a type of directed acyclic graph that includes nodes for indicators of missingness – is recommended when faced with missing data problems (Lee et al., 2023; Madley-Dowd et al., 2019). Selection and attrition bias can be addressed through inverse-probability weighting (Box 1).

Choosing methodological innovations among the presented will depend on the research goal, available sociodemographic information, and data structure. In practice, research on marginalized populations will need to address common methodological challenges applicable to all populations, as well as those challenges specifically linked to the population of interest, such as incomplete assessment of marginalization status. While not all challenges described here can be fully solved, raising awareness by pointing out considerations relevant to marginalization is a first step toward more inclusive research.

Regardless of methodological finesse, without more accurate assessment of possible marginalization status, for instance the assessment of self-reported ethnicity, migration status, assigned sex at birth as well as gender identity, methodological innovations will not be able to overcome limitations of data collection. It is therefore warranted that survey specialists are cognizant of the relevance of detailed sociodemographic assessment.

## Conclusion

Research on marginalized populations is fundamental to ensure an equitable distribution of healthcare and social care resources. Phenomenologically, we need to better understand commonalities and deviations in ageing of underrepresented groups compared to what is currently considered “normal” ageing, and possible intersectionalities of age, sex/gender, marginalization status, and other characteristics. Neuroscience methods should assess the unique biological manifestations of experiences of extreme marginalization linked to discrimination, trauma, violence, and armed conflict. While methodological innovations exist to optimize statistical inference and thus build new knowledge, efforts in data collection and increased data equity will be needed to facilitate these endeavors. Data collection efforts to improve data equity need to be strengthened globally.
